# Costs and Outcomes of Integrated Human African Trypanosomiasis Surveillance System Using Rapid Diagnostic Tests, Democratic Republic of the Congo

**DOI:** 10.3201/eid2708.202399

**Published:** 2021-08

**Authors:** Rian Snijders, Alain Fukinsia, Yves Claeys, Epco Hasker, Alain Mpanya, Erick Miaka, Filip Meheus, Marleen Boelaert

**Affiliations:** Swiss Tropical and Public Health Institute, Basel, Switzerland (R. Snijders);; University of Basel, Basel (R. Snijders); Institute of Tropical Medicine, Antwerp, Belgium (R. Snijders, Y. Claeys, E. Hasker, M. Boelaert);; Programme National de Lutte contre la Trypanosomiase Humaine Africaine, Kinshasa, Democratic Republic of the Congo (A. Fukinsia, A. Mpanya, E. Miaka);; International Agency for Research on Cancer, Lyon, France (F. Meheus)

**Keywords:** human African trypanosomiasis, sleeping sickness, surveillance, diagnosis, integration, primary health services, costs, parasites, Democratic Republic of the Congo

## Abstract

We integrated sleeping sickness case detection into the primary healthcare system in 2 health districts in the Democratic Republic of the Congo. We replaced a less field-friendly serologic test with a rapid diagnostic test, which was followed up by human African trypanosomiasis microscopic testing, and used a mixed costing methodology to estimate costs from a healthcare provider perspective. We screened a total of 18,225 persons and identified 27 new cases. Average financial cost (i.e., actual expenditures) was US $6.70/person screened and $4,464/case diagnosed and treated. Average economic cost (i.e., value of resources foregone that could have been used for other purposes) was $9.40/person screened and $6,138/case diagnosed and treated. Our study shows that integrating sleeping sickness surveillance into the primary healthcare system is feasible and highlights challenges in completing the diagnostic referral process and developing a context-adapted diagnostic algorithm for the large-scale implementation of this strategy in a sustainable and low-cost manner.

Sleeping sickness, or human African trypanosomiasis (HAT), is a neglected tropical disease that has killed thousands of persons in sub-Saharan Africa since the beginning of the 20th century. This disease is caused by *Trypanosoma brucei gambiens*e and *T. brucei rhodesiense* parasites. This article focuses on *T. brucei gambiense* infections, which account for >98% of all HAT cases (*1*). After intense control efforts during the colonial period, the disease subsided but reemerged in the 1970s and peaked in the 1990s, when >30,000 new cases were reported annually in 1997 and 1998. By the end of the 20th century, increased HAT control efforts reversed the epidemic trend (*2*). This success persuaded the World Health Organization (WHO) to target HAT for elimination as a public health problem by 2020 and to eliminate transmission by 2030 (*3*). In 2018, only 977 new HAT cases were reported globally, >75% of which occurred in the Democratic Republic of the Congo (DRC) (*2*,*4*).

HAT control activities consist of case detection and management complemented with vector control. Case detection can be done actively through outreach campaigns or passively by screening self-reporting cases in medical facilities. The passive approach accounted for >50% of the cases detected in DRC in 2017. With the declining prevalence, and therefore a higher cost of outreach activities on a per-case-found basis, passive screening might figure more prominently in future strategies for HAT elimination (*4*,*5*). Moreover, the past has shown that inadequate HAT surveillance can lead to reemerging epidemics, further underscoring the need for sustained epidemiologic surveillance and case detection in the general health system (*6*,*7*).

Historically, passive detection of HAT in DRC was conducted mainly at designated centers for HAT diagnosis, treatment, and control because of the complexity of diagnostic procedures. Clinical diagnosis of HAT is difficult because of its nonspecific symptoms in the early stages, and HAT needs to be confirmed because of the complex and toxic treatment regimens currently available. First, a relatively easy and cheap serologic screening test is performed, which, if positive, is followed by microscopic testing to confirm the presence of the parasite in the lymph fluid or blood. Then, a lumbar puncture is necessary to determine if the disease has advanced to the neurologic stage, given that, until 2019, the treatment regimen was different for cases in the hematolymphatic stage (stage 1) versus those in the meningoencephalitic stage (stage 2) (*1*,*8*,*9*).

Mitashi et al. (*5*) listed the preconditions for the integration of vertical disease control services as follows: a functional health system, versatile health workers, a minimum level of disease prevalence to maintain technical skills; decision-making powers for the health system combined with technical guidance by the disease program, and mutual benefits for the healthcare system and the disease program (*5*,*10*–*12*). This article examined 1 additional criterion, appropriate technology.

In the past, the main serologic test used for trypanosomiasis was the card agglutination test, which requires a rotator and a cold chain and is only available in 50 test dose vials with a limited shelf life once opened (1 week in a refrigerator or up to 8 hours at room temperature). The need for electric power combined with the high wastage given the low daily use, limits the usefulness of this test in first-line health services. In addition, microscopic examination to visualize the parasite requires specific laboratory skills and equipment (*5*,*13*). Recently, 2 rapid diagnostic tests (RDTs) for HAT became commercially available: the SD-Bioline HAT test (Abbot, https://www.globalpointofcare.abbott) and the HAT Sero-K-Set (Coris BioConcept, https://www.corisbio.com). These individual thermostable tests do not require equipment or cold storage and could improve the integration of case detection in the primary healthcare system (*14*). A study in Uganda demonstrated that RDTs would allow HAT screening to be integrated into the routine activities of health facilities (*15*,*16*). A comparison of HAT serologic tests showed that RDTs could be a cost-effective alternative to the card agglutination test in passive detection of trypanosomiasis at health facility level (*17*). Our study aimed to evaluate the results and costs of a HAT surveillance system that was based on RDTs, integrated into primary care facilities, and managed at the health district level.

## Methods

### Research Setting

Every province in the DRC is divided into health districts that consist of a network of health facilities that each serve a well-defined area of the district (*11*). The study took place in the HAT-endemic health districts of Mosango and Yasa Bonga in the former Bandundu Province in DRC. Both health districts together consist of 38 health areas, have a combined population of 369,393, and represent an area of 6,160 km^2^ (Yasa Bonga, 235,696 population and an area of 2,810 km^2^; Mosango, 133,697 population and an area of 3,350 km^2^) (*18*,*19*). During 2000–2012, a total of 45% of all HAT cases in DRC were reported in Bandundu Province, and during this period, the highest annual incidence reported in both health districts was 40 cases/10,000 population (*20*).

### Integrating HAT Case Detection and Management

During the preintervention phase, investments were made to strengthen the infrastructure, equipment, and staff skills before integrating HAT screening because the districts did not meet several integration requirements highlighted by Mitashi et al. (*5*). In addition, research showed that a poorly regulated fee-for-health services payment system could lead to unpredictable health costs for patients, which reduces access to quality healthcare (*9*). Therefore, a flat-rate payment system was introduced to improve financial access to healthcare in both districts.

**Figure 1:**
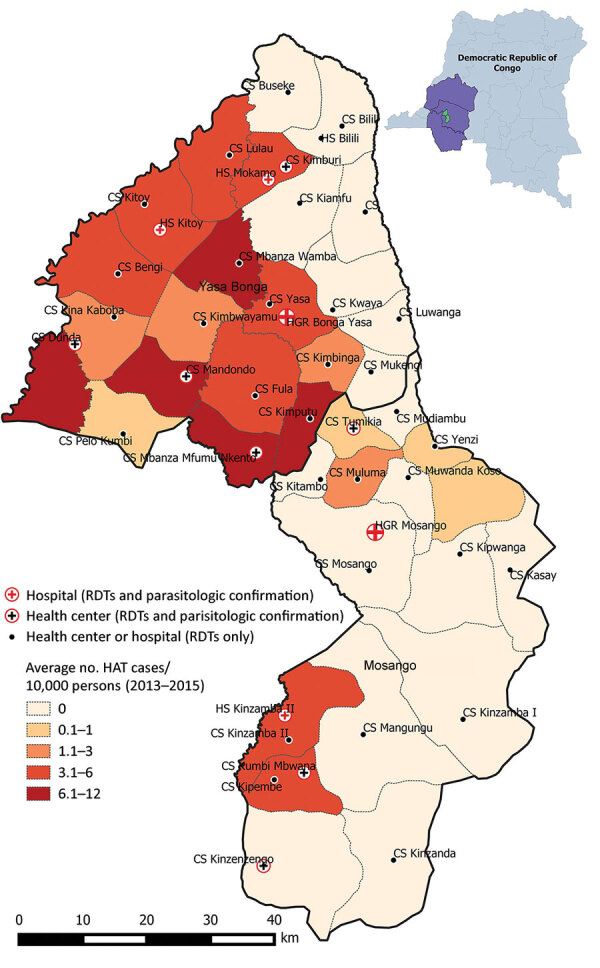
Health facilities performing HAT surveillance and the average human African trypanosomiasis incidence (cases/10,000 population), Democratic Republic of the Congo, 2013–2015. Inset shows location of the country in Africa. Map generated by using QGIS 3.10.1 (*4*). HAT, human African trypanosomiasis; RDT, rapid diagnostic test.

Before 2015, only 5 facilities in the study area were able to perform serologic and parasitologic tests. The intervention planned for serologic screening in >1 health facility per health area and the ability to perform HAT microscopic testing nearby. The facilities were chosen on the basis of HAT incidence during 2013–2015 and population density (Figure 1).

The intervention started with training staff and reinforcing HAT management skills at the health district level. The health district management teams and the experts from the national sleeping sickness control program (Programme National de Lutte contre la Trypanosomiase Humaine Africaine en République Démocratique Du Congo) oversaw training, management, and supply.

**Figure 2:**
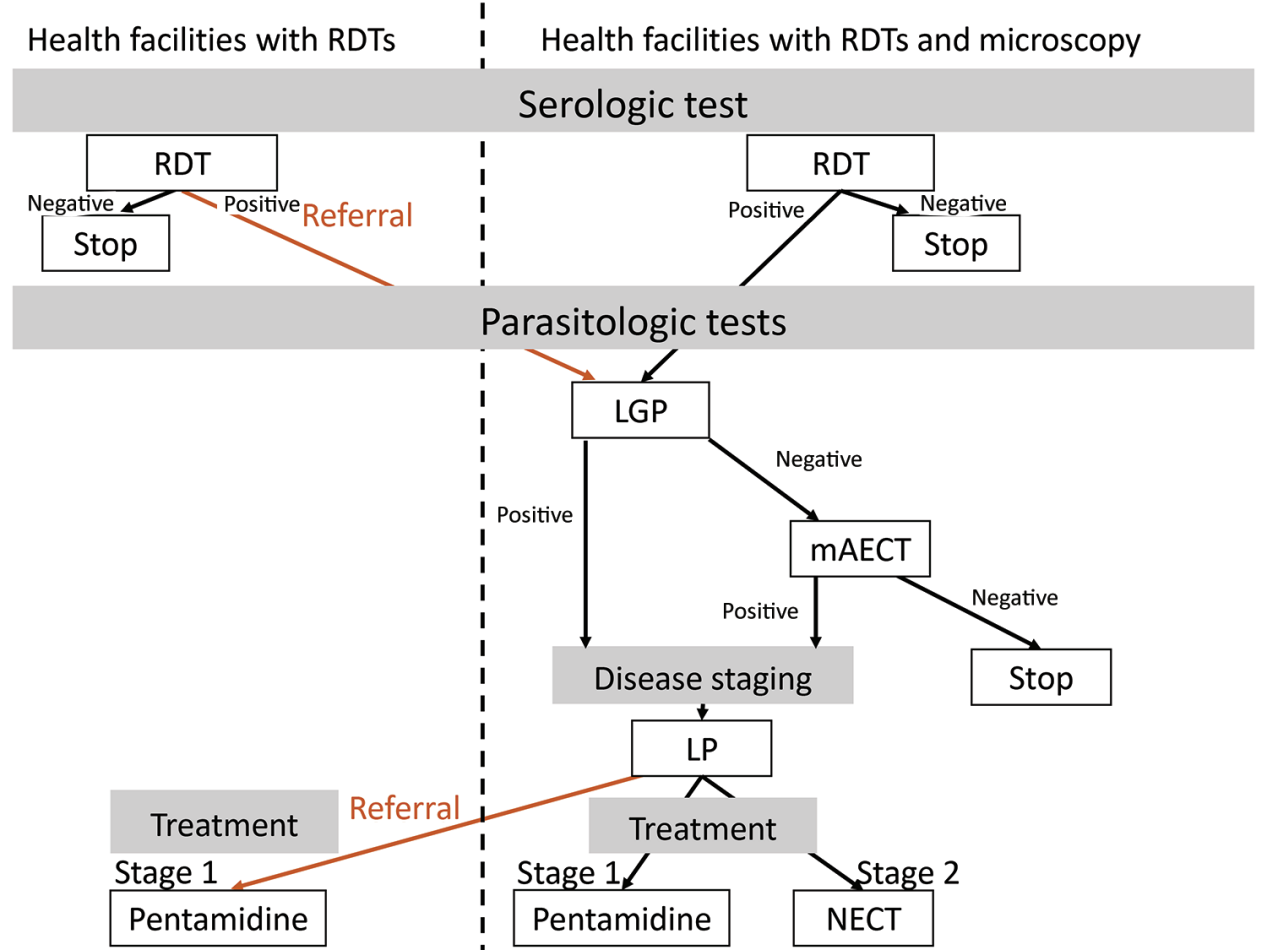
Diagnostic algorithm applied after a negative malaria test, persistent fever after malaria treatment, or symptoms suggestive of human African trypanosomiasis, Democratic Republic of the Congo. LGP, Lymph gland puncture; LP, lumbar puncture; mAECT, mini anion exchange centrifugation test; NEXT, nifurtimox/eflornithine combination therapy; RDT, rapid diagnostic test.

The screening algorithm indicated that all patients with a negative malaria test or persistent fever after a malaria treatment or >1 signs or symptoms suggestive of HAT (e.g., lymphadenopathy, headache, pruritus, musculoskeletal pain, hepatomegaly, splenomegaly, sleep disorder, and neuropsychiatric symptoms) were to be screened with a HAT RDT (*11*,*20*). HAT microscopic testing was to be conducted for all patients with a positive HAT RDT, either onsite or at the nearest facility with microscopic testing capacity (Figure 2). The microscopic testing consisted of a lymph gland puncture to examine the fluid for parasites if swollen glands were present, followed by the more sensitive mini anion exchange centrifugation test if no such glands were present or if the result of the lymph gland puncture was negative. Patients were considered to have a confirmed HAT case when trypanosomes were observed. The cerebrospinal fluid of patients was to be examined with a lumbar puncture because of the stage-specific treatment available at the time of the study, followed by treatment according to WHO and national guidelines (*21*–*24*). Stage 1 consisted of outpatient treatment with pentamidine at a health facility close to the patient’s home. Stage 2 consisted of inpatient treatment in a health facility qualified to administer nifurtimox/eflornithine combination therapy.

By the end of 2016, integrated HAT surveillance was operational. HAT screening with RDTs was available in 48 facilities, and microscopic diagnostic testing was available in 11 facilities (Table 1) (*25*).

**Table 1:** Number of facilities able to perform passive case detection of human African trypanosomiasis per health district before and after implementing the intervention, Democratic Republic of the Congo, 2017–2018 (*25*)

District and type of facility	Before the intervention			After the intervention	
	Serologic screening*	Parasitologic diagnosis†		Serologic screening	Parasitologic diagnosis
Mosango					
Hospital‡	1	1		2	2
Health center				17	2
Yasa Bonga					
Hospital	3	3		4	3
Health center	1	1		25	4
Total	5	5		48	11

*Sleeping sickness rapid diagnostic tests †Lymph gland puncture, mini anion exchange centrifugation test, and lumbar puncture. ‡Reference or secondary hospital.

### Data Collection and Analysis

We collected data during January 1, 2017–December 31, 2018. Data were based on operational and financial reports, field visits, and discussions with experts.

#### Number of Persons Screened, Diagnosed, and Treated

The primary indicator for measuring the output of both health districts was the number of persons screened for HAT and cases identified and treated. Of the 1,092 monthly reports expected during the study period from all participating health facilities, 91 reports (8%) were not retrieved. Most of the missing reports coincided with periods when HAT RDTs were out of stock. Therefore, we assumed that no HAT screening activities took place during the unreported months.

Because integrating of disease control services requires a functional healthcare system, we tracked the utilization rate for the health district by using the number of curative consultations annually per total population. The DRC’s national guidelines state that in a well-functioning health district, this rate should be >0.5 consultations/capita (*26*).

#### Financial and Economic Costs

We estimated economic and financial costs from the health provider’s perspective. Financial costs represent the actual expenditure, whereas economic costs estimate the value of resources foregone that could have been used for other purposes. Costs incurred by households, research costs, and costs of activities during the preintervention phase were not included.

We recorded all costs in the currency they were incurred and converted to US dollars (USD) based on the average exchange rate during the study period (Euro to USD, 1.15; Congolese Franc to USD, 0.00067). The costs exclude the DRC’s 16% value-added tax, from which the national program and donors are exempt (*27*). Transport and importation costs for goods that needed to be imported into DRC were estimated at 10% of the procurement cost on the basis of the average shipment costs between Belgium and DRC during the study period.

We used bottom-up microcosting to assess the cost of HAT tests and equipment. For capital equipment provided for HAT microscopic testing, we annualized the purchase or replacement value on the basis of the expected useful life of items and discounted them at a rate of 3% (*28*). We assigned a proportion of this cost to HAT testing on the basis of the expected proportion of time for which the equipment would be used for HAT tests. We estimated the cost of HAT testing by multiplying the number of persons tested by the average cost of all consumables used per test. During the study, we used only SD-Bioline HAT RDTs at a per-unit purchase price of $0.55. SD-Bioline receives a subsidy of $0.25 per test from a private donor. The per-unit price of the HAT RDT Sero-K-set was €1.79 (*17*).

The flat-rate payment system implemented a fixed consultation rate of 5,000 Congolese Francs (+ 3.35) that enables health facilities to recover their costs with an average estimated consultation time of 15 minutes. Performing an RDT takes ≈15–20 minutes. The patients did not pay any additional fees nor did the facilities receive any support besides the HAT tests and equipment. We included the consultation fee in the economic cost as a proxy to estimate the costs incurred by health facilities to provide the services (i.e., nurse time and use of facility resources) and the consultation fee was excluded from the financial cost estimate because no actual expenses were incurred.

For HAT treatment, we obtained outpatient follow-up and hospitalization costs from WHO and combined them with the cost for drugs used to treat side effects on the basis of the average costs of the medication during treatment in both districts in 2017. We included no HAT-specific treatment costs because pentamidine and nifurtimox/eflornithine combination therapy are donated by pharmaceutical companies (*29*–*33*).

We used top-down gross costing to estimate costs related to training and management. We annualized HAT training costs on the basis of the period between refresher training sessions. For the management costs, we included financial and in-kind support provided to the health facilities and management cost at provincial and health district level. We accounted for management costs of the national program at national level by applying a 15% markup on the activities managed by the program, which corresponds to the overhead rate the program applies for several projects to finance its role as national coordinator of HAT activities. The costs do not include transport costs of test or equipment from the capital city (Kinshasa) to the field because the districts were supplied during regular supervision visits. We estimated the cost per person screened and per case diagnosed and treated by dividing the overall cost of the intervention by the number of persons screened and treated.

#### Sensitivity Analysis

We used univariate sensitivity analysis to assess the impact of changes in the main cost drivers, such as the costs incurred to provide the services, including the cost of treatment and the price of RDTs. We also varied the discount rate between 0% and 5%.

### Ethics

Ethics approval for this study was obtained from the institutional review board of the Institute of Tropical Medicine in Antwerp, Belgium (approval no. IRB/AB/ac/137, protocol no. 115/16) and the institutional review board of School of Public Health of the University of Kinshasa, in Kinshasa, DRC (approval no. ESP/CE/08/2017). The study evaluated costs and aggregated operational data of routine activities provided by the healthcare system. Therefore, no formal consent was needed.

## Results

### Number of Persons Screened, Confirmatory Tested, Diagnosed, and Treated

Both health districts were considered well-functioning during the study period; the district utilization rate was close to the national threshold of 0.5 consultations per inhabitant per year (0.53 in Yasa Bonga and 0.44 in Mosango). In 2018, only 29% (36,363/125,674) of the overall curative consultations in Yasa Bonga were done in health facilities involved in HAT screening and 77% in Mosango (46,009/59,228) (*18*,*34*), meaning that higher coverage of passive HAT screening was reached in Mosango, and ≈70% of the curative consultations in Yasa Bonga took place in healthcare centers not participating in HAT screening or during periods when no HAT screening was reported. For both districts, >50% of the curative consultations involved testing with a malaria RDT, ≈60% of which tested positive.

In total, 18,225 persons were screened for HAT with a HAT RDT (i.e., ≈80% of persons that tested negative for malaria), of whom 223 [1.22%] tested positive. RDT stock-outs were the main reason that 20% of malaria-negative persons were not tested for HAT. No reports were found indicating that persons were screened for HAT on the basis of persistent fever after a malaria treatment or >1 signs or symptoms suggestive of HAT.

In total, 27 new HAT patients were identified through a positive mini anion exchange centrifugation test (no positive lymph gland puncture). Only 55% of the persons with a positive HAT RDT (123/223) were tested to confirm the presence of the parasite, because only 20% (25/122) of the persons with a positive HAT RDT identified in a facility without HAT microscopic testing available completed the referral. In comparison, 97% (98/101) of RDT-positive persons identified in facilities equipped to perform microscopic testing completed confirmation. Of the 27 new cases identified and treated in 2017 and 2018, a total of 9 were detected through healthcare centers and 18 by the reference and secondary hospitals (Appendix Tables 1–4).

### Financial Costs

The total annual financial cost for both health districts was US $123,386 in 2017 and $28,710 in 2018; the average annual financial cost over 5 years was $62,500. The higher financial cost in the first year is attributable to staff training and equipment purchases. The financial cost is substantially lower than the economic cost because it does not consider any support for human resources or the use of other resources for the health facilities performing the tests (Appendix Table 5).

### Economic Costs

We constructed an overview of the economic costs by input and activity (Table 2). The total economic cost in Mosango is ≈5% higher than in Yasa Bonga because >30% more persons were screened, leading to higher facility and RDT costs. The higher cost in Mosango is partly offset by the lower training costs, because fewer facilities were involved in HAT screening than in Yasa Bonga (Appendix Tables 6–17).

**Table 2:** Annual economic costs of passive human African trypanosomiasis screening in Mosango and Yasa Bonga health districts, Democratic Republic of the Congo*

Cost category and subcategory	Description	Cost, USD			Total, %
		Mosango	Yasa Bonga	Total	
Capital equipment		18,008	25,051	43,060	25
Equipment	Confirmation equipment	4,734	6,627	11,360	7
	Laboratory equipment	2,539	3,554	6,093	4
	Nonmedical equipment	2,195	3,073	5,268	3
Training	HAT diagnosis training	13,275	18,424	31.699	19
	Screening	5,079	6,950	12,029	7
	Microscopy	8,196	11,474	19,670	12
Annual recurrent costs		69,243	56,764	126,008	75
Laboratory and medical supplies	HAT tests	7,487	5,449	12,262	7
	RDTs	7,388	4,874	12,262	7
	Microscopy	99	575	673	0.4
Patient care	Staging and inpatient and outpatient care	413	1,601	2,014	1
HR and infrastructure use	Execution RDT	36,535	24,102	60,637	36
Management	Management and supervision	24,808	25,613	50,421	30
Total		87,251	81,816	169,067	100
Cost per person screened		7.95	11.29	9.28	
Cost per case diagnosed and treated		21,813	3,557	6,262	

*HAT, human African trypanosomiasis; HR, human resources; RDT, rapid diagnostic test.

#### Economic Cost Per Person Screened and Per Case Diagnosed and Treated

**Figure 3:**
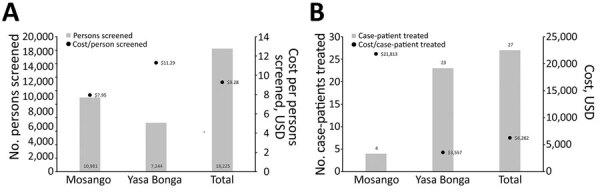
Cost per person screened and per human African trypanosomiasis case diagnosed and treated, Democratic Republic of the Congo.

The overall cost per person screened was $9.28, and the cost per case diagnosed and treated was $6,262 (Figure 3). In Yasa Bonga, the cost per person screened is higher than in Mosango because of the higher number of facilities involved and the lower number of persons screened. However, the average cost per case diagnosed and treated is much lower in Yasa Bonga because of the higher number of cases detected and treated.

### Sensitivity Analysis

**Figure 4:**
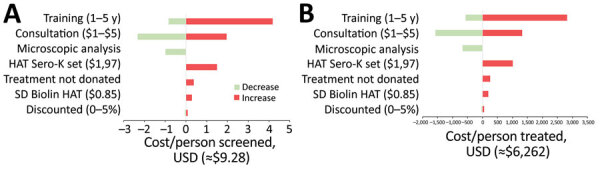
Sensitivity analysis on main cost drivers for HAT diagnosis and treatment, Democratic Republic of the Congo. HAT, human African trypanosomiasis.

We summarized the results of the univariate sensitivity analysis of several cost parameters to assess the potential impact on the cost per person screened and cost per case diagnosed and treated (Figure 4). The main cost drivers are the frequency of training and the cost at health facility level to provide this service. The economic cost per person screened or case diagnosed would be much lower if we assume that the health system can provide HAT screening by using fewer additional resources than those needed for a 15-minute consultation (the proxy used to estimate the cost at health facility level, including human resources and infrastructure); however, this approach might overestimate the health system’s capacity. A lower estimated unit cost to provide this service of $1 instead of $3.55 would lower the cost per person screened and diagnosed and treated by 25%. Further, the study assumed that healthcare workers needed retraining every 3 years. Increasing the frequency of the laboratory technicians’ training increases the cost per person screened and diagnosed and treated by 45%. Reducing the number of facilities where HAT microscopic testing is available decreases the cost per person screened and diagnosed and treated. Using more expensive tests or treatments increases the cost per person. Varying the discount rate from 0% to 5% had little effect on the cost estimates.

## Discussion

This study describes the development, implementation, and cost of a strategy for HAT case detection integrated into the primary healthcare system in DRC using a novel screening test. In a context of a declining number of cases combined with a need for sustained surveillance, policymakers need to reflect on the value of integrating HAT screening into the basic health service package offered by polyvalent first-line health services. Introducing HAT RDTs helped integrate HAT screening into the primary healthcare system in both health districts where the program waspiloted. Although the number of persons screened almost doubled, the number of cases identified declined, consistent with the observed overall decrease in prevalence in both districts. This decline resulted in an increased cost per person diagnosed and treated. The cost per person diagnosed and treated through passive screening estimated in this study is much higher than the cost per HAT case cured reported in an earlier study that evaluated the cost-effectiveness of screening for HAT with RDTs ($6,262 compared with $278, or $10,133/36.5 cases) (*17*). The earlier study modeled the use of HAT RDTs in a high-volume hospital that screened 2,500 patients annually for HAT and detected 36.5 HAT cases, whereas, in our study in 2018, the average number of persons screened per facility was 206 (9,892 persons/48 facilities), and the average number of cases detected per facility, 0.6 (27 cases/48 facilities), therefore incurred higher fixed facility-level costs (capital and district supervision) for services to fewer patients.

Furthermore, training and management costs were not included in previous studies, and the estimated cost of the use of the facilities’ resources was much lower ($1 vs. $3.33 per person screened). The cost per person screened through passive screening in the study area is much higher than through active screening ($9.28 vs. an average of $2.1). However, the average cost per case detected is much lower ($6,318 vs. an average of $16,080) because of the higher proportion of cases detected in the population screened during passive screening than during active screening (*35*).

**Figure 5:**
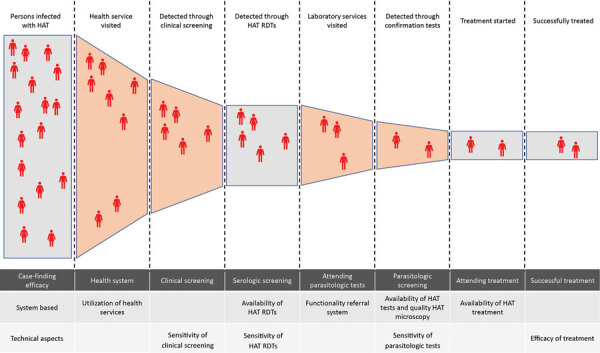
Illustration of potential loss in effectiveness in passive screening for HAT integrated into the primary healthcare system using an adaptation of Piot model for tuberculosis (*36*), Democratic Republic of the Congo. HAT, human African trypanosomiasis; RDT, rapid diagnostic test.

The effectiveness of this strategy should be evaluated through the number of HAT cases detected and treated. Several potential bottlenecks were identified in the process of HAT case detection (*36*) (Figure 5). The main challenges in the study area were the fact that potential HAT cases were not detected because the person had already tested positive on a malaria RDT or because they did not complete the referral for offsite microscopic testing. Today, a novel therapeutic, fexinidazole, has obviated the need for staging in a portion of patients and could improve the effectiveness of this system; however, there are several contraindications against this treatment (*37*).

The following recommendations should be considered for the scale-up of passive surveillance through RDTs. The HAT screening algorithm should be context-specific, a negative malaria test in a malaria-endemic area might not be a good preselection criterion for HAT screening. Furthermore, the system should be adapted to demand (e.g., it should be located in facilities that are most frequented, exploit the existing referral system to supply HAT test material, and take into account a minimum attendance rate). In the study setting, a separate referral and supply system for HAT was set up and closely monitored by the national program. Shifting most of these tasks to the general healthcare system will probably lower the cost and render the system more sustainable when implemented on a larger scale. The shift in service delivery might also cause a shift in the financing of the system. In this study, the costs at health facility level were borne by the health facilities because they did not receive any additional compensation for the extra time spent on HAT testing, which is reflected in the lower financial cost. Health facilities might be reluctant to participate in HAT activities without any in-kind or financial compensation. A remedy might be to include HAT formally into the performance-based financing system.

In conclusion, HAT case detection and surveillance integrated into the primary healthcare system using RDT’s showed promising results but also substantial practical weaknesses. Integration is possible in a sustainable and low-cost manner if challenges regarding completing diagnostic algorithm are addressed and a context-adapted diagnostic algorithm is used.

Appendix Additional information about costs and outcomes of integrated human African trypanosomiasis surveillance system using rapid diagnostic tests, Democratic Republic of the Congo.
