# Supplementary material for: Costs and Outcomes of Integrated Human African Trypanosomiasis Surveillance System Using Rapid Diagnostic Tests, Democratic Republic of the Congo
Source: Emerg Infect Dis. 2021 Aug;27(8):2144–53. doi: 10.3201/eid2708.202399 (PMC8314840; doi:10.3201/eid2708.202399)
Supplement: Appendix — Additional information about costs and outcomes of integrated human African trypanosomiasis surveillance system using rapid diagnostic tests, Democratic Republic of the Congo. [file 20-2399-Techapp-s1.pdf]

Article DOI: https://doi.org/10.3201/eid2708.202399

# Costs and Outcomes of Integrated Human African Trypanosomiasis Surveillance System Using Rapid Diagnostic Tests, Democratic Republic of the Congo

## Appendix

## Materials and Methods

### Function used to estimate the annual financial cost

Total annual financial cost excluding variable management cost (Total annual cost excluding variable management cost x 1.15) =

Purchase/replacement value capital equipment and training

+ People screened x cost Rapid Diagnostic test (Cost for the RDT implementation at health facility level is not taken in account because this is not directly financed by the health care provider)

+ People microscopically confirmed x (cost Blood sample + cost mAECT)

+ Cases x cost lumbar puncture (LP)

+ Stage 1 cases x Cost treatment with pentamidine

+ Stage 2 cases x Cost treatment with NECT

+ Costs for management and supervision

Total annual cost = Total annual cost excluding variable management cost x 1.15

### Function used to estimate the annual economic cost

Total annual economic cost excluding variable management cost =

Annualized discounted cost capital equipment and training

+ People screened x cost Rapid Diagnostic test

+ People screened x cost RDT implementation at health facility level

+ People microscopically confirmed x (cost Blood sample (BS) + cost mAECT) (None of the facilities reported any lymph node aspirations done and no cases were diagnosed through lymph node aspirations.)

+ Cases x cost lumbar puncture (LP)

+ Stage 1 cases x Cost treatment with pentamidine

+ Stage 2 cases x Cost treatment with NECT

+ Costs for management and supervision

Total annual economic cost = Total annual economic cost excluding variable management cost x 1.15

#### Function used to estimate the annualized discounted economic cost of capital equipment

The annualised discounted economic costs of the equipment was calculated by averaging the annual discounted costs based on the useful life of the equipment. For each year (n) in the future the value of costs was multiplied by $1/(1+D)^n$ where D is the discount rate.

For example for a car with a value of 40,000$ with a useful life of 4 years and a discount rate of 3% the annual discounted economic cost would be calculated as followed:

$[10,000 * (1/(1+0.03)^0+1/(1+0.03)^1+1/(1+0.03)^2+/(1+0.03)^3)]/4$

[10,000 * (1+0.97+0.94+0.92)]/4 = 38,286 /4 = 9,571

**Appendix Table 1.** Number of curative consultations, malaria tests and HAT tests performed, and HAT cases detected

| Characteristic | Curative consultations | Number of malaria RDTs performed | Malaria RDT negative | Number of HAT RDTs performed | HAT RDT positive | Number of mAECT performed | mAECT positive/ HAT cases |
|---|---|---|---|---|---|---|---|
| Mosango | 91,053 | 56,266 | 12,823 | 10,981 | 81 | 18 | 4 |
| Facilities: RDT & mAECT | 17,591 | 6,849 | 1,190 | 1,320 | 14 | 11 | 0 |
| Facilities: RDT only | 73,463 | 49,417 | 11,634 | 9,661 | 67 | 7 | 4 |
| Yasa Bonga | 63,435 | 37,452 | 9,189 | 7,244 | 142 | 105 | 23 |
| Facilities: RDT & mAECT | 20,051 | 9,829 | 3,686 | 3,106 | 87 | 87 | 18 |
| Facilities: RDT only | 43,384 | 27,623 | 5,503 | 4,138 | 55 | 18 | 5 |
| Grand Total | 154,488 | 93,718 | 22,012 | 18,225 | 223 | 123 | 27 |

**Appendix Table 2.** Number of confirmation test done on sleeping sickness seropositives (n = 223)*

| | | Results microscopy tests | | | | | |
|---|---|---|---|---|---|---|---|
| Characteristic | Not tested | mAECT neg | mAECT pos; Stage 1 | mAECT pos; Stage 2 | mAECT pos; Stage unknown | Total | Total mAECT pos. |
| Screened at RDT centers | 97 (80%) | 16 (13%) | 2 (2%) | 5 (4%) | 2 (2%) | 122 | 9 (36%) |
| Screened at RDT & mAECT centers | 3 (3%) | 80 (79%) | 1 (1%) | 16 (16%) | 1 (1%) | 101 | 18 (8%) |
| Total screened | 100 (45%) | 96 (43%) | 3 (1%) | 21 (9%) | 3 (1%) | 223 | 27 (22%) |

*Total mAECT positives as a proportion of total mAECT tested.

**Appendix Table 3.** Disease stage* of HAT cases identified in the study facilities in each health district*

| | Mosango | | | Yasa Bonga | | | Total | | |
|---|---|---|---|---|---|---|---|---|---|
| Characteristic | 2017 | 2018 | Total | 2017 | 2018 | Total | 2017 | 2018 | Total |
| Stage 1 | 0 | 0 | 0 | 8 | 3 | 11 | 8 | 3 | 11 |
| Stage 2 | 2 | 0 | 2 | 7 | 4 | 11 | 9 | 4 | 13 |
| Stage unknown | 2 | 0 | 2 | 0 | 1 | 1 | 2 | 1 | 3 |
| Grand Total | 4 | 0 | 4 | 15 | 8 | 23 | 19 | 8 | 27 |

*Stage 1: hematolymphatic stage of the disease; Stage 2: meningoencephalitic stage of the disease.

**Appendix Table 4.** Number of curative consultations, malaria tests and HAT tests performed, and HAT cases detected per health facility in 2017 and 2018

| | Curative consultations | | | RDT Malaria | | | RDT Malaria + | | | RDT HAT | | | RDT HAT + | | | mAECT | | | New cases | | |
|---|---|---|---|---|---|---|---|---|---|---|---|---|---|---|---|---|---|---|---|---|---|
| Characteristic | 2017 | 2018 | Total | 2017 | 2018 | Total | 2017 | 2018 | Total | 2017 | 2018 | Total | 2017 | 2018 | Total | 2017 | 2018 | Total | 2017 | 2018 | Total |
| Mosango | 45,045 | 46,009 | 91,053 | 27,966 | 28,300 | 56,266 | 21,173 | 22,270 | 43,442 | 5,725 | 5,256 | 10,981 | 57 | 24 | 81 | 9 | 9 | 18 | 4 | - | 4 |
| RDT & mAECT | 12,077 | 10,236 | 22,313 | 5,464 | 4,062 | 9,526 | 4,229 | 3,331 | 7,560 | 1,328 | 757 | 2,085 | 17 | 8 | 25 | 7 | 6 | 13 | 2 | | 2 |
| CS KINZENZENGO | 4,532 | 3,214 | 7,746 | 1,673 | 1,901 | 3,574 | 1,309 | 1,688 | 2,997 | 670 | 281 | 951 | 6 | 2 | 8 | | 1 | 1 | | | |
| CS KUMBI MBWANA | 3,879 | 1,787 | 5,666 | 3,252 | 1,249 | 4,501 | 2,573 | 1,027 | 3,600 | 269 | 223 | 492 | 2 | 1 | 3 | | 1 | 1 | | | |
| HGR MOSANGO | 2,444 | 3,154 | 5,598 | 287 | 334 | 621 | 135 | 149 | 284 | 266 | 48 | 314 | 3 | - | 3 | 3 | | 3 | | | |
| HS/CSR KINZAMBA II | 1,222 | 2,081 | 3,303 | 252 | 578 | 830 | 212 | 467 | 679 | 123 | 205 | 328 | 6 | 5 | 11 | 4 | 4 | 8 | 2 | | 2 |
| RDT | 32,968 | 35,773 | 68,741 | 22,502 | 24,238 | 46,740 | 16,944 | 18,939 | 35,883 | 4,397 | 4,499 | 8,896 | 40 | 16 | 56 | 2 | 3 | 5 | 2 | - | 2 |
| CS Mosenge (KINZAMBA II) | 4,362 | 3,712 | 8,074 | 1,657 | 2,114 | 3,771 | 1,195 | 1,181 | 2,376 | 617 | 482 | 1,099 | 5 | - | 5 | | | | | | |
| CS Camp Pompe/CS Mosango | 3,152 | 3,910 | 7,062 | 2,812 | 2,779 | 5,591 | 2,039 | 2,256 | 4,295 | 771 | 456 | 1,227 | 1 | - | 1 | | | | | | |
| CS KASAY | 3,133 | 806 | 3,939 | 2,270 | 1,153 | 3,423 | 1,804 | 840 | 2,644 | 401 | 325 | 726 | 5 | - | 5 | 1 | | 1 | 1 | | 1 |
| CS KINZAMBA I | 2,852 | 2,508 | 5,360 | 1,737 | 1,624 | 3,361 | 1,422 | 1,288 | 2,710 | 212 | 271 | 483 | 1 | - | 1 | 1 | | 1 | 1 | | 1 |
| CS MUDIAMBU | 2,628 | 3,190 | 5,818 | 2,084 | 2,448 | 4,532 | 1,427 | 1,937 | 3,364 | 480 | 501 | 981 | 17 | 4 | 21 | | | | | | |
| CS MULUMA | 2,506 | 3,061 | 5,567 | 1,480 | 1,559 | 3,039 | 1,217 | 1,298 | 2,515 | 76 | 59 | 135 | 1 | 2 | 3 | | | | | | |

| | Curative consultations | | | RDT Malaria | | | RDT Malaria + | | | RDT HAT | | | RDT HAT + | | | mAECT | | | New cases | | |
|---|---|---|---|---|---|---|---|---|---|---|---|---|---|---|---|---|---|---|---|---|---|
| Characteristic | 2017 | 2018 | Total | 2017 | 2018 | Total | 2017 | 2018 | Total | 2017 | 2018 | Total | 2017 | 2018 | Total | 2017 | 2018 | Total | 2017 | 2018 | Total |
| CS YENZI | 2,461 | 2,295 | 4,756 | 1,806 | 1,336 | 3,142 | 1,498 | 1,087 | 2,585 | 336 | 284 | 620 | 1 | 1 | 2 | | | | | | |
| CS KITAMBO | 2,436 | 2,523 | 4,959 | 2,027 | 1,887 | 3,914 | 1,555 | 1,595 | 3,150 | 275 | 238 | 513 | 1 | - | 1 | | | | | | |
| CS MUWANDA KOSO | 2,362 | 2,125 | 4,487 | 1,765 | 1,399 | 3,164 | 1,363 | 1,129 | 2,492 | 311 | 257 | 568 | - | - | - | | | | | | |
| CS KIPWANGA | 2,277 | 2,947 | 5,224 | 1,824 | 2,121 | 3,945 | 1,284 | 1,684 | 2,968 | 273 | 397 | 670 | 4 | - | 4 | | | | | | |
| CS MANGUNGU | 2,105 | 1,649 | 3,754 | 1,152 | 958 | 2,110 | 683 | 734 | 1,417 | 295 | 187 | 482 | 1 | 1 | 2 | | | | | | |
| CS KIPEMBE | 1,657 | 2,252 | 3,909 | 1,288 | 1,689 | 2,977 | 1,008 | 1,398 | 2,406 | 266 | 229 | 495 | 3 | 6 | 9 | | 2 | 2 | | | |
| CS KINZANDA | 1,037 | 1,205 | 2,242 | 600 | 871 | 1,471 | 449 | 751 | 1,200 | 84 | 133 | 217 | - | - | - | | | | | | |
| CS Mbulu | | 1,443 | 1,443 | | 1,019 | 1,019 | | 709 | 709 | | 494 | 494 | | 1 | 1 | | 1 | 1 | | | |
| CS Kumbi Makopa | | 2,147 | 2,147 | | 1,281 | 1,281 | | 1,052 | 1,052 | | 186 | 186 | | 1 | 1 | | | | | | |
| Yasa Bonga | 27,072 | 36,363 | 63,435 | 16,933 | 20,519 | 37,452 | 12,037 | 16,226 | 28,263 | 2,608 | 4,636 | 7,244 | 69 | 73 | 142 | 40 | 65 | 105 | 15 | 8 | 23 |
| RDT & mAECT | 10,590 | 11,391 | 21,981 | 5,780 | 5,529 | 11,309 | 3,320 | 3,824 | 7,144 | 1,175 | 2,347 | 3,522 | 36 | 60 | 96 | 31 | 60 | 91 | 12 | 6 | 18 |
| HGR BONGA YASA | 1,978 | 1,932 | 3,910 | 717 | 726 | 1,443 | 217 | 335 | 552 | 319 | 1,249 | 1,568 | 4 | 17 | 21 | 4 | 17 | 21 | 2 | 3 | 5 |
| HS KITOY | 2,417 | 1,160 | 3,577 | 1,054 | 550 | 1,604 | 615 | 343 | 958 | 94 | 155 | 249 | 17 | 12 | 29 | 17 | 12 | 29 | 7 | 3 | 10 |
| HS MOKAMO | 2,260 | 2,283 | 4,543 | 1,040 | 814 | 1,854 | 386 | 361 | 747 | 316 | 475 | 791 | 6 | 12 | 18 | 6 | 12 | 18 | 3 | | 3 |
| CS MBANZA MFUMU NKENTO | 1,419 | 2,487 | 3,906 | 1,057 | 1,390 | 2,447 | 777 | 1,079 | 1,856 | 124 | 138 | 262 | 1 | 4 | 5 | | 4 | 4 | | | |
| CS DUNDA | 1,101 | 1,646 | 2,747 | 788 | 913 | 1,701 | 538 | 683 | 1,221 | 113 | 168 | 281 | 5 | 4 | 9 | 4 | 4 | 8 | | | |
| CS KIMPUTU | 853 | 697 | 1,550 | 670 | 373 | 1,043 | 484 | 328 | 812 | 133 | 82 | 215 | 2 | 3 | 5 | | 3 | 3 | | | |
| CS MANDONDO | 562 | 1,186 | 1,748 | 454 | 763 | 1,217 | 303 | 695 | 998 | 76 | 80 | 156 | 1 | 8 | 9 | | 8 | 8 | | | |
| RDT | 16,482 | 24,972 | 41,454 | 11,153 | 14,990 | 26,143 | 8,717 | 12,402 | 21,119 | 1,433 | 2,289 | 3,722 | 33 | 13 | 46 | 9 | 5 | 14 | 3 | 2 | 5 |
| CS YASA | 1,335 | 2,093 | 3,428 | 765 | 1,272 | 2,037 | 605 | 1,072 | 1,677 | 80 | 191 | 271 | 5 | - | 5 | 1 | | 1 | | | |
| CS KWAYA | 1,235 | 1,288 | 2,523 | 848 | 776 | 1,624 | 704 | 644 | 1,348 | 59 | 122 | 181 | 1 | 1 | 2 | 1 | | 1 | | | |
| CS KIMBWAYAMU | 987 | 942 | 1,929 | 706 | 623 | 1,329 | 561 | 507 | 1,068 | 108 | 92 | 200 | 4 | 1 | 5 | 3 | 1 | 4 | 1 | | 1 |
| CS LULAU | 982 | 1,236 | 2,218 | 669 | 816 | 1,485 | 565 | 718 | 1,283 | 105 | 94 | 199 | 2 | - | 2 | | | | | | |
| CS MBANZA GOBARI | 974 | 946 | 1,920 | 760 | 769 | 1,529 | 646 | 600 | 1,246 | 76 | 95 | 171 | 4 | - | 4 | | | | | | |
| CS LUWANGA | 969 | 1,010 | 1,979 | 601 | 704 | 1,305 | 403 | 539 | 942 | 119 | 132 | 251 | - | 1 | 1 | | 1 | 1 | | | |
| CS FULA | 814 | 2,096 | 2,910 | 656 | 749 | 1,405 | 529 | 689 | 1,218 | 104 | 54 | 158 | 5 | 1 | 6 | | | | | | |
| CS PELO KUMBI | 673 | 759 | 1,432 | 378 | 373 | 751 | 303 | 290 | 593 | 28 | 85 | 113 | 1 | 1 | 2 | | | | | | |
| CS KIMBINGA | 966 | 1,255 | 2,221 | 525 | 803 | 1,328 | 322 | 585 | 907 | 39 | 194 | 233 | 1 | - | 1 | | | | | | |
| CS KIAMFU | 941 | 965 | 1,906 | 627 | 621 | 1,248 | 477 | 450 | 927 | 66 | 201 | 267 | 1 | - | 1 | 1 | | 1 | | | |
| CS KINA KABOBA | 912 | 883 | 1,795 | 631 | 718 | 1,349 | 513 | 645 | 1,158 | 53 | 123 | 176 | 1 | - | 1 | 1 | | 1 | | | |
| CS BUSEKE | 885 | 978 | 1,863 | 665 | 494 | 1,159 | 508 | 418 | 926 | 128 | 76 | 204 | - | - | - | | | | | | |
| CS MBANZA WAMBA | 867 | 862 | 1,729 | 750 | 733 | 1,483 | 633 | 631 | 1,264 | 135 | 115 | 250 | 1 | - | 1 | | | | | | |
| CS BENGI | 814 | 1,387 | 2,201 | 445 | 975 | 1,420 | 397 | 949 | 1,346 | 39 | 46 | 85 | 2 | 3 | 5 | | 1 | 1 | | 1 | 1 |
| CS BILILI | 801 | 903 | 1,704 | 542 | 635 | 1,177 | 447 | 578 | 1,025 | 40 | 63 | 103 | 4 | - | 4 | 2 | | 2 | 2 | | 2 |
| CS KIMBURI | 733 | 993 | 1,726 | 461 | 392 | 853 | 253 | 293 | 546 | 68 | 63 | 131 | - | 1 | 1 | | 1 | 1 | | | |
| CS KITOY | 760 | 532 | 1,292 | 460 | 320 | 780 | 369 | 285 | 654 | 48 | 23 | 71 | 1 | 2 | 3 | | 1 | 1 | | 1 | 1 |
| HS BILILI | 498 | 699 | 1,197 | 391 | 629 | 1,020 | 254 | 433 | 687 | 93 | 203 | 296 | - | - | - | | | | | | |
| CS MUKENGI | 336 | 883 | 1,219 | 273 | 607 | 880 | 228 | 515 | 743 | 45 | 115 | 160 | - | 2 | 2 | | | | | | |
| CS MBANZA NGANDA | | 1,199 | 1,199 | | 448 | 448 | | 391 | 391 | | 49 | 49 | | - | - | | | | | | |
| CS MATAMBA | | 1,212 | 1,212 | | 661 | 661 | | 573 | 573 | | 82 | 82 | | - | - | | | | | | |
| CS KISANGANI | | 1,851 | 1,851 | | 872 | 872 | | 597 | 597 | | 71 | 71 | | - | - | | | | | | |
| Grand Total | 72,117 | 82,372 | 154,488 | 44,899 | 48,819 | 93,718 | 33,210 | 38,496 | 71,705 | 8,333 | 9,892 | 18,225 | 126 | 97 | 223 | 49 | 74 | 123 | 19 | 8 | 27 |

**Appendix Table 5.** Annual financial costs of integrated passive screening in Yasa Bonga and Mosango

| | | Based on the average 2017 - 2018 | | | | | | |
|---|---|---|---|---|---|---|---|---|
| Assumptions | Activity | 2017 | 2018 | *Year 3* | *Year 4* | *Year 5* | | |
| Number of people screened annually | | 8,333 | 9,892 | 9,113 | 9,113 | *9,113* | | |
| People tested through microscopy | | 49 | 74 | 123 | 123 | *123* | | |
| Number of people treated stage 1 | | 8 | 3 | 6 | 6 | *6* | | |
| Number of people treated stage 2 | | 11 | 5 | 8 | 8 | *8* | | |
| **Description** | | **2017** | **2018** | **Year 3** | **Year 4** | ***Year 5*** | | |
| Capital Equipment | | 83,613 $ | 810 $ | 2,958 $ | 61,771 $ | *2,958 $* | | |
| Medical and laboratory equipment | Microscopy | 13,074 $ | 810 $ | 2,958 $ | 810 $ | *2,958 $* | | |
| Data collection equipment | Microscopy | 9,578 $ | - $ | - $ | - $ | *- $* | | |
| Solar panel (energy source) | Microscopy | 12,000 $ | - $ | - $ | 12,000 $ | *- $* | | |
| Training – Screening | Training RDT | 18,579 $ | | | 18,579 $ | | | |
| Training - Parasitological confirmation | Training mAECT | 30,382 $ | | | 30,382 $ | | | |
| Annual Recurrent costs | | 39,773 $ | 27,900 $ | 27,970 $ | 36,792 $ | *27,970 $* | | |
| Lab & medical supplies | RDT | 5,606 $ | 6,655 $ | 6,131 $ | 6,131 $ | *6,131 $* | | |
| Lab & medical supplies | Microscopy | 268 $ | 405 $ | 339 $ | 339 $ | *339 $* | | |
| Lab & medical supplies | Staging | 359 $ | 151 $ | 264 $ | 264 $ | *264 $* | | |
| RDT implementation at health facility level | Health facility | | | | | | | |
| Treatment (Hospitalization & drugs) | Treatment | 1,040 $ | 464 $ | 759 $ | 759 $ | *759 $* | | |
| Health District level: Management, Support & Supervision (MOH) | Management | 6,760 $ | 6,760 $ | 6,760 $ | 6,760 $ | *6,760 $* | | |
| Provincial Health Division: Supervision (MOH) | Management | 420 $ | 420 $ | 420 $ | 420 $ | *420 $* | | |
| Provincial Coordination: Management, Support & Supervision (PNLTHA) | Management | 7,664 $ | 7,664 $ | 7,664 $ | 7,664 $ | *7,664 $* | Average | |
| Central Coordination: Management, Support & Supervision (PNLTHA) | Management | 17,655 $ | 5,381 $ | 5,632 $ | 14,454 $ | *5,632 $* | 3 y | 5 y |
| Total | | 123,386 $ | 28,710 $ | 30,928 $ | 98,563 $ | *30,928 $* | 61,008 $ | 62,503 $ |
| Cost per person screened | | 13.54 $ | 3.15 $ | 3.39 $ | 10.82 $ | *3.39 $* | 6.69 $ | 6.86 $ |
| Cost per person treated | | 8,813 $ | 2,051 $ | 2,209 $ | 7,040 $ | *2,209 $* | 4,464 $ | 4,529 $ |

**Appendix Table 6.** Total annual economic costs of integrated passive screening in Yasa Bonga and Mosango

| | | 2017 | 2017 | 2018 | 2018 | 2017-2018 | 2017-2018 | 2017-2018 |
|---|---|---|---|---|---|---|---|---|
| Description | Activity | Mosango | Yasa Bonga | Mosango | Yasa Bonga | Mosango | Yasa Bonga | Total |
| Capital Equipment | | 9,004 $ | 12,526 $ | 9,004 $ | 12,526 $ | 18,008 $ | 25,051 $ | 43,060 $ |
| Medical and laboratory equipment | Microscopy | 1,269 $ | 1,777 $ | 1,269 $ | 1,777 $ | 2,539 $ | 3,554 $ | 6,093 $ |
| Data collection equipment | Microscopy | 693 $ | 970 $ | 693 $ | 970 $ | 1,386 $ | 1,940 $ | 3,326 $ |
| Solar panel (energy source) | Microscopy | 405 $ | 567 $ | 405 $ | 567 $ | 809 $ | 1,133 $ | 1,942 $ |
| Training – Screening | Training RDT | 2,539 $ | 3,475 $ | 2,539 $ | 3,475 $ | 5,079 $ | 6,950 $ | 12,029 $ |
| Training - Parasitological confirmation | Training mAECT | 4,098 $ | 5,737 $ | 4,098 $ | 5,737 $ | 8,196 $ | 11,474 $ | 19,670 $ |
| Annual Recurrent costs | | 35,795 $ | 24,341 $ | 33,448 $ | 32,424 $ | 69,243 $ | 56,764 $ | 126,008 $ |
| Lab & medical supplies | RDT | 3,852 $ | 1,755 $ | 3,536 $ | 3,119 $ | 7,388 $ | 4,874 $ | 12,262 $ |
| Lab & medical supplies | Microscopy | 49 $ | 219 $ | 49 $ | 356 $ | 99 $ | 575 $ | 673 $ |
| Lab & medical supplies | Staging | 76 $ | 283 $ | - $ | 151 $ | 76 $ | 434 $ | 510 $ |
| RDT implementation at health facility level | Health facility | 19,048 $ | 8,677 $ | 17,487 $ | 15,425 $ | 36,535 $ | 24,102 $ | 60,637 $ |
| Treatment (Hospitalization & drugs) | Treatment | 337 $ | 703 $ | - $ | 464 $ | 337 $ | 1,167 $ | 1,504 $ |
| Health District level: Management, Support & Supervision (MOH) | Management | 3,380 $ | 3,380 $ | 3,380 $ | 3,380 $ | 6,760 $ | 6,760 $ | 13,520 $ |
| Provincial Health Division: Supervision (MOH) | Management | 210 $ | 210 $ | 210 $ | 210 $ | 420 $ | 420 $ | 840 $ |
| Provincial Coordination: Management, Support & Supervision (PNLTHA) | Management | 3,832 $ | 3,832 $ | 3,832 $ | 3,832 $ | 7,664 $ | 7,664 $ | 15,328 $ |
| Central Coordination: Management, Support & Supervision (PNLTHA) | Management | 5,012 $ | 5,282 $ | 4,953 $ | 5,487 $ | 9,964 $ | 10,769 $ | 20,734 $ |

| Description | Activity | 2017 Mosango | 2017 Yasa Bonga | 2018 Mosango | 2018 Yasa Bonga | 2017-2018 Mosango | 2017-2018 Yasa Bonga | 2017-2018 Total |
|---|---|---|---|---|---|---|---|---|
| Total | | | 36,866 $ | 42,452 $ | 44,949 $ | 87,251 $ | 81,816 $ | 169,067 $ |
| Cost per person screened | | 7.83 $ | 14.14 $ | 8.08 $ | 9.70 $ | 7.95 $ | 11.29 $ | 9.28 $ |
| Cost per person treated | | 11,200 $ | 2,458 $ | NA | 14,983 $ | 21,813 $ | 3,557 $ | 6,262 $ |

**Appendix Table 7.** Economic cost - Capital equipment: Detailed costs of capital equipment for HAT microscopy tests per health facility ($)

| Category | Description | No. | Replacement Value excl VAT | Useful life | % of Use allocated to HAT | Annual cost | Annual cost Discounted at 3% | Annual cost Discounted at 5% | Information Source |
|---|---|---|---|---|---|---|---|---|---|
| Medical and laboratory eq. | Microscope incl. accessories - 12V | 1 | 843 | 5 | 25% | 42 $ | 40 $ | 38 $ | Invoice2017 |
| Medical and laboratory eq. | Centrifuge - 12V | 1 | 179 | 2 | 100% | 90 $ | 88 $ | 87 $ | Invoice2017 |
| Medical and laboratory eq. | Holder mAECT | 1 | 9 | 1 | 100% | 9 $ | 9 $ | 9 $ | Invoice2018 |
| Medical and laboratory eq. | Reading Chamber mAECT | 2 | 58 | 1 | 100% | 117 $ | 117 $ | 117 $ | Invoice2019 |
| Data collection equipment | PDA & camera incl. accessories | 1 | 798 | 5.4 | 100% | 148 $ | 139 $ | 133 $ | Invoice2017 |
| Solar panel (energy source) | Solar panel incl. accessories | 1 | 1,000 | 3 | 25% | 83 $ | 81 $ | 79 $ | Invoice2017 |
| Total | | | | | | 489 $ | 473 $ | 464 $ | |

**Appendix Table 8.** Economic cost - Capital equipment: Detailed training costs: HAT awareness, use HAT RDTs, use PDA

| Description | Unit cost ($) | Quantity | Total ($) |
|---|---|---|---|
| Public transport to health zones | 30 | 23 | 675 |
| Per diem participants | 20 | 90 | 1,800 |
| Per diem personnel supporting staff coordination | 10 | 12 | 120 |
| Housing Participants & supporting staff coordination | 20 | 102 | 2,040 |
| Lunch & coffee breaks | 15 | 102 | 1,530 |
| Meeting room | 100 | 4 | 400 |
| Fuel generator 1l/h = >7l/day | 1.40 | 28 | 39 |
| Office supplies | 15 | 23 | 338 |
| Printing training module | 10 | 23 | 225 |
| Other costs | 750 | 1 | 750 |
| Per diem Central level PNLTHA | 85 | 12 | 1,020 |
| Fuel −21l/100km - 1100km retour Kinshasa + 100km circulation | 1.40 | 252 | 353 |
| Total | | | 9,290 |
| | | Total cost per structure | 413 |
| | | Discounted cost (3%) – estimated lifespan 3 y | 401 |
| | | Discounted cost (5%) – estimated lifespan 3 y | 394 |

**Appendix Table 9.** Economic cost - Capital equipment: Detailed training costs: HAT microscopy tests

| Description | Unit cost ($) | Quantity | Total ($) |
|---|---|---|---|
| Public transport health zones | 30 | 12 | 360 |
| Per diem participants | 20 | 90 | 1,800 |
| Per diem supporting staff coordination | 10 | 12 | 120 |

| Description | Unit cost ($) | Quantity | Total ($) |
|---|---|---|---|
| Housing participants and supporting staff coordination | 20 | 210 | 4,200 |
| Lunch & coffee breaks | 15 | 210 | 3,150 |
| Meeting room | 50 | 14 | 700 |
| Fuel generator 1L/hour, or 7L/day | 1.40 | 28 | 39 |
| Office supplies | 15 | 12 | 180 |
| Printing training module | 10 | 12 | 120 |
| Training equipment (mice, etc.) | 1,500 | 1 | 1,500 |
| Other costs | 500 | 1 | 500 |
| Per diem Central level PNLTHA & INRB | 85 | 56 | 4,760 |
| Fuel central level −21L/100km - 1100km round-trip; to Kinshasa + 100km | 1.40 | 252 | 353 |
| Total | | | 17,782 |
| | | Total cost per structure | 1,482 |
| | | Discounted cost (3%) - estimated lifespan 3 y | 1,439 |
| | | Discounted cost (5%) - estimated lifespan 3 y | 1,412 |

**Appendix Table 10.** Economic cost - Capital equipment: Detailed training costs internships of microscopists with a mobile team

| Description | Unit cost ($) | Quantity | Total ($) |
|---|---|---|---|
| Transport Kwilu, Kwango | 50 | 3 | 150 |
| Allowance/screening tour (10 $/30 d) | 300 | 3 | 900 |
| Total | | | 1,050 |
| | | Total cost per structure | 1,050 |
| | | Discounted cost (3%) - estimated lifespan 3 y | 1,020 |
| | | Discounted cost (5%) - estimated lifespan 3 y | 1,001 |

**Appendix Table 11.** Economic cost - Annual recurrent costs: Detailed costs per test*

| Description | Packaging | # Units/ packaging | Price | Currency | Unit price ($) | Import | Unit price in Kinshasa ($) | LGP | BS | mAECT | LP | RDT |
|---|---|---|---|---|---|---|---|---|---|---|---|---|
| Cotton balls | Roll | 1 | 7.00 | $ | 7.00 | | 7.00 | | | | | |
| Providone - disinfectant | 250 ml | 1 | 5.00 | $ | 5.00 | | 5.00 | | | | | |
| Gloves | Box | 100 | 7.00 | $ | 0.07 | | 0.07 | | 1 | | | 1 |
| Bin | Piece | 1 | 20.00 | $ | 20.00 | | 20.00 | | | | | |
| Kit CATT | Kit CATT | 1 | 0.52 | Euro | 0.61 | x | 0.67 | | | | | |
| Lancet | Box | 200 | 3.10 | Euro | 0.02 | x | 0.02 | | | | | |
| Heparinized capillary tubes | Box | 100 | 3.03 | Euro | 0.04 | x | 0.04 | | | | | |
| Bulb for capillary tubes | Box | 100 | 1.42 | Euro | 0.02 | x | 0.02 | | | | | |
| Hypodermic needle | Box | 100 | 8.00 | $ | 0.08 | | 0.08 | 1 | | | | |
| Syringe 5cc | Box | 100 | 7.00 | $ | 0.07 | | 0.07 | 1 | | | 1 | |
| Tropicalized microscope slide | Box | 50 | 4.00 | $ | 0.08 | | 0.08 | 1 | | | | |
| Cover glass | Box | 100 | 2.00 | $ | 0.02 | | 0.02 | 1 | | | | |
| Gauze | Box | 10 | 3.50 | $ | 0.35 | | 0.35 | | 0.5 | | | |
| Adaptor vacutainer tubes | Box | 1 | 0.50 | $ | 0.50 | | 0.50 | | 0.5 | | | |
| Vacutainer needle | Box | 1 | 0.50 | $ | 0.50 | | 0.50 | | 1 | | | |
| Heparinized vacutainer tubes | Box | 100 | 35.00 | $ | 0.35 | | 0.35 | | 1 | | | |

| Description | Packaging | # Units/ packaging | Price | Currency | Unit price ($) | Import | Unit price in Kinshasa ($) | LGP | BS | mAECT | LP | RDT |
|---|---|---|---|---|---|---|---|---|---|---|---|---|
| Plasticine | Sheet | 6 | 30.00 | $ | 5.00 | | 5.00 | | | | | |
| Specialized cover glass | Box | 10 | 15.00 | $ | 1.50 | | 1.50 | | | | 1 | |
| Kit mAECT | Box | 1 | 3.50 | Euro | 4.13 | | 4.13 | | | 1 | | |
| Lumbar puncture needle | Piece | 1 | 1.30 | $ | 1.30 | | 1.30 | | | | 1 | |
| Modified single centrifugation kit | Kit | 1 | 10.00 | Euro | 11.80 | | 11.80 | | | | 1 | |
| Collector tube mAECT (price = Kit) | Box | 1 | 3.50 | Euro | 4.13 | | 4.13 | | | | 1 | |
| Pipette | Box | 500 | 45.00 | $ | 0.09 | | 0.09 | | | | 1 | |
| Tips | Box | 500 | 4.24 | Euro | 0.01 | x | 0.01 | | | | | |
| Microtitration tray | Box | 50 | 20.01 | Euro | 0.47 | x | 0.52 | | | | | |
| RDT standard diagnostics | Box | 25 | 13.70 | $ | 0.55 | x | 0.60 | | | | | 1 |
| RDT HAT Sero-K-Set | Box | 40 | 60.80 | Euro | 1.79 | x | 1.97 | | | | | 1 |

*The costs per test was based on the observations regarding the consumables used during active screening activities and market prices during the project.

**Appendix Table 12.** Economic cost - Annual recurrent costs: Detailed Cost per test

| Test | Price subsidized | Price unsubsidized |
|---|---|---|
| Lymph node aspiration (LGP) | $ 0.25 | NA |
| Blood sample (BS) | $ 1.35 | NA |
| mAECT | $ 4.13 | $ 8.26 |
| Lumbar puncture examination (LP) | $18.89 | NA |
| RDT Standard Diagnostics | $ 0.67 | $ 0.92 |
| RDT Sero-K-Set | $ 1.97 | NA |

**Appendix Table 13.** Economic cost - Annual recurrent costs: Detailed costs per treatment

| Description | Value ($) | Min ($) | Max ($) | Source of information |
|---|---|---|---|---|
| Cost per day hospitalized | 1.64 | 1.25 | 2.23 | (*1*) |
| Cost per outpatient visit by hospital level* | 0.40 | 0.29 | 0.60 | (*1*) |
| Cost pentamidine | - | | 20.00 | Donated (*2*), |
| Number of days outpatient treatment with pentamidine | 10 | | | (*3*) |
| Cost other drugs administered during treatment with pentamidine | 10 | | | Observation patient charts Yasa Bonga & Mosango |
| Number of days hospitalized during treatment with NECT | 10 | | | (*4*) |
| Cost NECT | - | | 407 | Donated (*4*), |
| Cost other drugs administered during treatment with NECT | 10 | | | Observation patient charts Yasa Bonga & Mosango |
| Other costs related to a treatment with NECT | 58 | | 58 | (*5*) |

**Appendix 14.** Economic cost - Annual recurrent costs: Cost treatment

| Total cost per treatment | Value | Minimum | Maximum |
|---|---|---|---|
| Treatment stage 1 - Pentamidine | 14 | 3 | 36 |
| Treatment stage 2 - NECT | 84 | 13 | 497 |

**Appendix Table 15.** Economic cost - Annual recurrent costs: Detailed costs for management and supervision: Provincial coordination PNLTHA*

| Description | Annual cost ($) | Cost/HD** ($) |
|---|---|---|
| Global annual budget provincial coordination | 67,961 | 2,265 |
| Annual planning meeting at provincial level (ECP, ECZS, UM) | 13,475 | 449 |
| 1 supervision/Semester (2 people - 5 d/mission) | 1,117 | 1,117 |
| Total Provincial Level: Management & Supervision/HZ | | 3,832 |

*The coordination of Bandundu Sud dedicates around 30% of their time to passive screening in 18 endemic health districts (HD). Therefore, the estimate amount of their annual budget to be dedicated to passive screening per health zone is 3%. HD, health district.

**Appendix Table 16.** Economic cost - Annual recurrent costs: Detailed costs for management and supervision: National coordination PNLTHA*

| Description | Annual cost ($) | Cost/HD ($) |
|---|---|---|
| Management team to support former Bandundu coordination | 31,800 | 265 |
| National Level: 1 supervision/3 y - 3 people - 5 d | 1,432 | 1,432 |
| Total Central Level: Total direct costs | | 2,227 |
| Total Central Level: Total indirect costs | | 15% |

*Estimates based on the annual costs, budgets and interviews with PNLTHA. The project management team at the central level follows up HAT control activities in the coordinations of Bandundu Nord and Bandundu Sud. We estimated the team spends 2.5% of their time per health district and conducts one supervisory field visit every 3 y. For the PNTLHA management cost at central level a percentage of 15% on the activities managed by the PNLTHA is included.

**Appendix Table 17.** Economic cost - Annual recurrent costs: Detailed costs for management and supervision: Health district management unit and provincial health authorities*

| Description | Annual cost ($) | Cost/HD ($) |
|---|---|---|
| Health district level: Management, Support & Supervision | 3,380 | 3,380 |
| Provincial level - DPS: Management, Support & Supervision - 20 endemic health zones Kwilu | 4,200 | 210 |

*The cost for the HDM and DPS are based on the financial and in-kind support they received throughout the study period.

## References

1. World Health Organization. Estimates of unit costs for patient services for Democratic Republic of the Congo [cited 2019 Oct 12]. https://www.who.int/choice/country/cod/cost/en

2. Lutumba P, Makieya E, Shaw A, Meheus F, Boelaert M. Human African trypanosomiasis in a rural community, Democratic Republic of Congo. Emerg Infect Dis. 2007;13:248–54. PubMed https://doi.org/10.3201/eid1302.060075

3. Babokhov P, Sanyaolu AO, Oyibo WA, Fagbenro-Beyioku AF, Iriemenam NC. A current analysis of chemotherapy strategies for the treatment of human African trypanosomiasis. Pathog Glob Health. 2013;107:242–52. PubMed https://doi.org/10.1179/2047773213Y.0000000105

4. Simarro PP, Franco J, Diarra A, Postigo JA, Jannin J. Update on field use of the available drugs for the chemotherapy of human African trypanosomiasis. Parasitology. 2012;139:842–6. PubMed https://doi.org/10.1017/S0031182012000169

5. Yun O, Priotto G, Tong J, Flevaud L, Chappuis F. NECT is next: implementing the new drug combination therapy for *Trypanosoma brucei gambiense* sleeping sickness. PLoS Negl Trop Dis. 2010;4:e720-e. **PMID 20520803**
